# Synthesis and Spectroscopic Analyses of New Polycarbonates Based on Bisphenol A-Free Components

**DOI:** 10.3390/polym13244437

**Published:** 2021-12-17

**Authors:** Krystyna Wnuczek, Andrzej Puszka, Beata Podkościelna

**Affiliations:** Department of Polymer Chemistry, Faculty of Chemistry, Institute of Chemical Sciences, Maria Curie-Skłodowska University, Gliniana 33, 20-614 Lublin, Poland; andrzej.puszka@umcs.pl (A.P.); beata.podkoscielna@mail.umcs.pl (B.P.)

**Keywords:** polycarbonates, transesterification, polycondensation

## Abstract

This paper discusses a new synthesis of bisphenol A-free polycarbonates based on four aliphatic–aromatic systems. In the first stage, different types of monomers (with/without sulfur) derived from diphenylmethane were synthesized. Then, new polycarbonates were prepared in the reactions with diphenyl carbonate (DPC) by transesterification and polycondensation reactions. Three different catalysts (zinc acetate, 4-(dimethylamino)pyridine and benzyltriethylammonium chloride) were tested. The structures of the compounds were confirmed by Nuclear Molecular Resonance spectroscopy (NMR) in each stage. The chemical structures of the obtained polycarbonates were verified by means of Attenuated Total Reflectance Fourier Transform infrared spectroscopy (ATR–FTIR). The presence of a carbonyl group in the infrared spectrum confirmed polycarbonate formation. Thermal studies by differential scanning calorimetry (DSC) were carried out to determine the melting temperatures of the monomers. A gel permeation chromatography analysis (GPC) of the polycarbonates was performed in order to investigate their molar masses. Thermal analysis proved the purity of the obtained monomers; the curves showed a characteristic signal of melting. The obtained polycarbonates were characterized as having high resistance to organic solvents, including tetrahydrofuran. The GPC analysis proved their relatively large molar masses and their low dispersity.

## 1. Introduction

Polycarbonates are materials encountered in everyday life. They are a class of thermoplastic polymers that were formally esters of carbonic acid. They are characterized by numerous advantages such as good hardness, ductility, rigidity, transparency, and toughness and excellent mechanical properties [1]. Compared to other thermoplastics, the most advantageous properties are as follows: high impact strength, good dielectric properties, good dimensional stability, wide operating temperature range, high creep strength, small water absorption, and a self-extinguishing tendency. In addition, most of the polycarbonates are non-toxic, very hard, abrasion-resistant, and chemical-resistant materials [2,3].

Bisphenol A polycarbonate (BPA-PC), derived from petroleum, is one of the most important and widely commercialized polycarbonates. However, bisphenol A is a toxic compound that can induce chronic toxicity and environmental problems [4]. BPA-PC is the carbonic acid polyester derived from 2,2-bis(4-hydroxyphenyl) propane. This is the best known polycarbonic resin of this type because of its good mechanical, thermal, and electrical properties, as well as being made from available raw materials. Most of the commercially available polycarbonates have been synthesized using bisphenol A. This compound is a precursor of important plastics, primarily of some polycarbonates and epoxy resins [5]. The use of bisphenol A as a diol for the synthesis of polycarbonates is controversial. The detection of bisphenol A in the environment and food products has been the subject of much recent research. Some studies prove that the thermal treatment of any food packaging product containing bisphenol A causes it to be released into the food. Studies proved that at temperatures above 70 °C and at high humidity, polycarbonate is hydrolyzed to bisphenol A [6,7,8,9,10].

The conventional phosgene process for polycarbonate preparation has been eliminated because of the toxicity of phosgene. Historically, polycarbonates were obtained by the polycondensation of phosgene with aromatic diols [11]. More and more often, there are reports in the literature which describe other “phosgene-free” methods of synthesizing this group of polymers, based mainly on the transesterification of appropriate diols (mainly butane-1,4-diol and bisphenol A) and dimethyl or diphenyl carbonate.

In recent years a safe and environmentally favorable process for the synthesis of polycarbonates has been a research goal [12]. Diphenyl carbonate (DPC) and bisphenol A are the main raw materials for polycarbonate synthesis. The non-phosgene route became popular for polycarbonate synthesis through the melt transesterification of DPC and BPA [13,14,15]. Diphenyl carbonate is a sustainable and environmentally benign reagent mainly used as a phosgene substitute for the synthesis of polycarbonates. The transesterification of bisphenol A and diphenyl carbonate and ring-opening polymerization of macrocyclic oligomers are commonly known routes [16]. As the transesterification is achieved through melt polymerization, the solvent is not necessary. However, so far there is no reliable process to produce DPC from phenol without the use of phosgene. The synthesis of macrocyclic oligomers is essentially the same reaction as the production of PC from bisphenol A and phosgene. In this context, the wholly non-phosgene process to produce PC would be a very desirable alternative [17].

There are many entries in the literature regarding the transesterification of diols followed by polycondensation to polycarbonates. Kim and Lee compared the transesterification of bisphenol A with that of diphenyl carbonate or dimethyl carbonate to obtain polycarbonate precursors. They also used the direct oxidative carbonylation of bisphenol A (with carbon monoxide) to obtain polycarbonate precursors for the synthesis of phosgene-free polycarbonates. They concluded that the melt transesterification of bisphenol A and diphenyl carbonate occurred readily to produce reactive precursors without a significant equilibrium constraint [18]. On the other hand, these methods were used to obtain high-molecular-weight polymers. Park and co-workers have published a synthesis procedure to obtain polycarbonates with a molecular weight of 100,000−200,000 Da. In the first step, oligomers were formed bearing almost equal numbers of hydroxyl and methyl carbonate end-groups. In the second step, the condensation reaction was conducted at a high temperature to connect the −OH and –O–C(O)–OCH_3_ chain-ends while removing the generated methanol under reduced pressure [19]. Sun and Kucling have described the synthesis of high-molecular-weight polycarbonates based on organo-catalysis. However, in most cases, the use of metal-based catalysts is required for the preparation of aliphatic polycarbonates by the polycondensation method, which are difficult to remove completely from the final polymer [20]. Their study was focused on the synthesis of high-molecular-weight aliphatic polycarbonates using organo-catalysts via a two-step polycondensation of dimethyl carbonate and a linear alkane diol as monomers. In our paper, three catalysts were used and all of them were organic compounds.

With the growing concern about environmental pollution and global warming, developing eco-friendly materials has become a key global necessity. The main goal of this study was to develop a new method of obtaining polycarbonate materials so that their syntheses fit into the ideals of green chemistry.

The aim of this research was to synthesize new polymeric compounds based on safe (BPA-free), non-toxic components. A method of synthesizing aliphatic–aromatic compounds based on diphenylmethane has been developed. Twelve new polycarbonates based on diols or dithiol with the DPC monomer were obtained. Polycarbonates were prepared by the polycondensation reaction and their chemical structures were confirmed by means of ATR–FTIR. Their properties were examined by DSC analysis. Briefly, our method applied the DPC reagent to introduce carbonate groups. The transesterification reaction of DPC with diols/dithiol in the melt phase was performed, resulting in the formation of PC precursors. In the next step, PC compounds are amenable to the condensation polymerization reaction. The polycondensation step occurs under vacuum conditions to remove the phenol produced as a recyclable byproduct. The precursors obtained by this route have phenyl carbonate and/or hydroxy end-groups [18,21]. As diols/dithiol components, the diphenylmethane derivatives were used. The chemical structures of monomers were confirmed by NMR (and the ATR–FTIR analysis in the Supplementary Materials). For the obtained polycarbonates, ATR–FTIR and GPC were performed. A detailed study concerning the thermal analysis of the obtained polycarbonates (thermogravimetry and DSC) will be discussed in the next paper.

## 2. Materials and Methods

### 2.1. Chemicals

Diphenyl carbonate, methane dichloride, and tetrahydrofuran were purchased from Merck (Merck, Darmstadt, Germany). Catalysts: zinc acetate, 4-(dimethylamino)pyridine (DMAP) and benzyltriethylammonium chloride also were obtained from Merck (Merck, Darmstand, Germany). Purified water was delivered by Millipore (Millipore, UMCS Lublin, Poland).

### 2.2. Methods

The ^1^H and ^13^C NMR spectra were recorded using a Bruker Avance 300 MSL instrument (Bruker, Coventry, United Kingdom) operating at 500 MHz for ^1^H and 75 MHz for ^13^C resonance frequency. Chemical shifts were referenced to deuterated chloroform (CDCl_3_), which served as an internal standard. The coupling constants (J) are given in Hz. The abbreviations for signal patterns are as follows: s, singlet; d, doublet; t, triplet; q, quartet; m, multiplet; b, broad.

The attenuated total reflection (ATR) was recorded using infrared Fourier transform spectroscopy (ATR-FTIR) on a TENSOR 27, Bruker spectrometer, equipped with a diamond crystal (Germany). The spectra were recorded in the range of 600–4000 cm^−1^ with 64 scans per spectrum at a resolution of 4 cm^−1^.

Differential scanning calorimetry (DSC) curves were obtained with the use of a DSC Netzsch 204 calorimeter (Netzsch, Günzbung, Germany). All DSC measurements were made using aluminum pans with pierced lids sample mass of 5–10 mg in a nitrogen atmosphere (30 mL/min). As the reference, an empty aluminum crucible was used. Dynamic scans were made at a heating rate of 10 K/min. The heating cycle was in the temperature range 0–200 °C. Parameters such as melting temperature (T_m_) and enthalpy of melting (ΔH_m_) were also determined.

The number ($\bar{M}$_n_), weight ($\bar{M}$_w_), average molar mass (g/mol), and molar mass dispersity (Ð_M_) of the obtained polycarbonates were determined by gel permeation chromatography (GPC) performed on a Viscotek GPC max (Viscotek, Kennesaw, USA) equipped with the triple detector array TDA 305. The eluent was tetrahydrofuran (THF), the flow rate was 1 mL/min, the operation temperature was set to 35 °C, and the molar mass was calibrated with polystyrene standards.

### 2.3. Synthesis of Monomers

**Figure 1:**
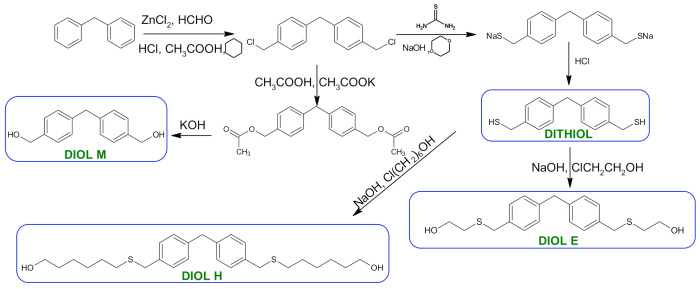
Scheme of monomers synthesis.

The syntheses of monomers were carried out according to the methods developed in the Department of Polymer Chemistry, UMCS. The reaction scheme is presented in Figure 1. The syntheses of diols E and H and dithiol have been reported in the literature [22,23]. The description of the syntheses of these three monomers can be found in the Supplementary Materials (S2.3). A detailed description of the synthesis of the diol M is provided below.

#### Synthesis of (methanediyldibenzene-4,1-diyl)dimethanol (diol M)

In total, 78 g (0.37 mol) of 1,1-methanediylbis[4-(chloromethyl)benzene] and 77 g (0.78 mol) of potassium acetate were weighed and added into a 1000 cm^3^ round-bottom flask. Boiling pebbles and 600 cm^3^ of pure acetic acid were added. The contents of the flask were boiled gently for 6 h. In order to avoid the sediment sticking to the flask, the whole construction was shaken. The solution was then decanted into a beaker and the KCl remained in the flask. An amount of 1 L of distilled water was added to the solution. An oil crystallized while stirring and was separated. The product was filtered off using a funnel and, after being transferred to a flask, 300 cm^3^ of 10% aqueous Na_2_CO_3_ solution was poured over it, and then it was filtered again in the funnel. The resulting product was transferred to a 1000 cm^3^ flask and treated with 5% aqueous KOH solution (100 cm^3^) and 650 cm^3^ of methanol. The flask was heated under reflux. The state of boiling was maintained for 3 h. Then, the alcohol was distilled off. The separated precipitate was filtered off using a funnel and then washed with distilled water until reaching neutral pH. The crude product was purified by crystallization from ethyl acetate (1 g per 7 cm^3^ of the solvent).

### 2.4. Synthesis of Polymers

**Figure 2:**
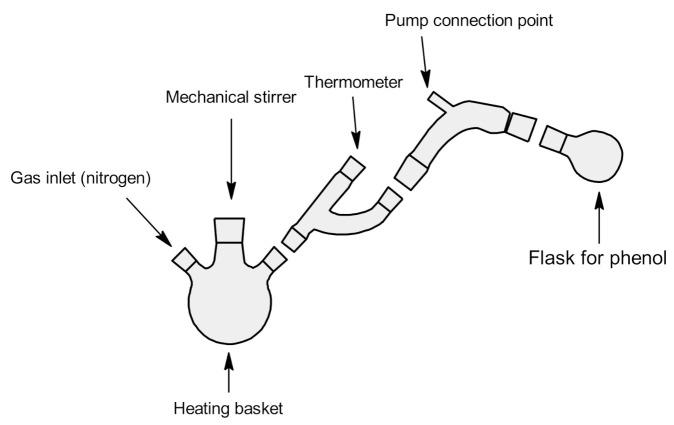
Diagram of laboratory glass used in the synthesis of polycarbonates.

**Figure 3:**
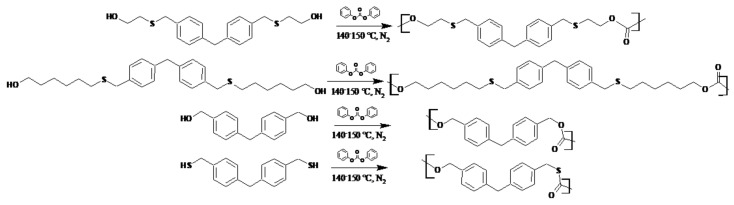
Scheme of syntheses of polycarbonates.

**Figure 4:**
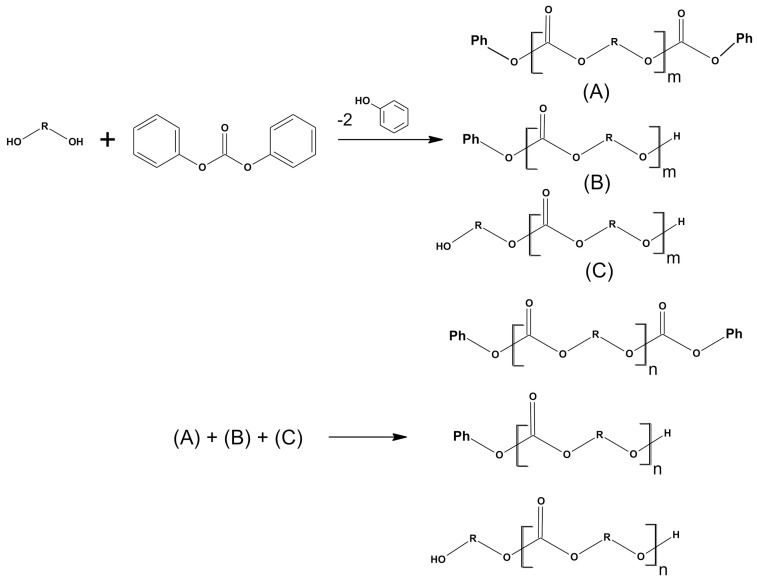
The probable course of the transesterification and polycondensation reactions with the use of diols and DPC [24]. (**A**), (**B**), (**C**): Oligomers.

The synthesis method is based on two consecutive reactions. In the first stage, the transesterification reaction takes place (with simultaneous removal of the phenol byproduct from the reaction medium), and in the next stage, polycondensation takes place. For the production of polymers, diphenyl carbonate was selected as a monomer introducing carbonate groups (for the production of aliphatic–aromatic polycarbonates). Diphenyl carbonate and one of the monomers were placed in a three-necked flask equipped with a mechanical stirrer, gas inlet, thermometer and water pump. Substrates were added in a stoichiometric ratio of 1:1. The catalyst was added in an amount equivalent to 0.1 mol%. The reactions were conducted in a nitrogen atmosphere for 2 h after the complete melting of the components. The temperature was kept at 140–150 °C. As the reaction proceeded, phenol was released, which was fed by a water pump to the flask. After two hours, the gas supply and the water pump were disconnected. Reactions were carried out for another 2 h under a vacuum pump, maintaining the temperature at 140–150 °C. The resulting polycarbonates were placed into beakers, treated with dichloromethane (50 mL), and precipitated with methanol. Then, they were left to evaporate the solvent. For complete drying of polycarbonates, the beakers were placed in an oven for 12 h (50 °C). Figure 2 presents the laboratory glass diagram. Figure 3 and Figure 4 show the schemes of reactions and probable reaction courses.

**Figure 5:**
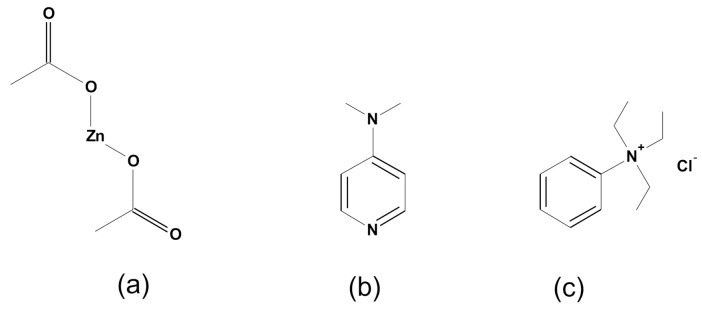
Catalysts: (**a**) zinc acetate, (**b**) DMAP, and (**c**) benzyltriethylammonium chloride.

Three types of catalysts were used in the reactions. Their structural formulae are listed in Figure 5. In total twelve parallel reactions were performed: each of the four monomers with three catalysts. Twelve products and twelve comparative materials (after precipitation with methanol) were obtained.

## 3. Results and Discussion

### 3.1. ^1^H and ^13^C NMR Analysis

**Figure 6:**
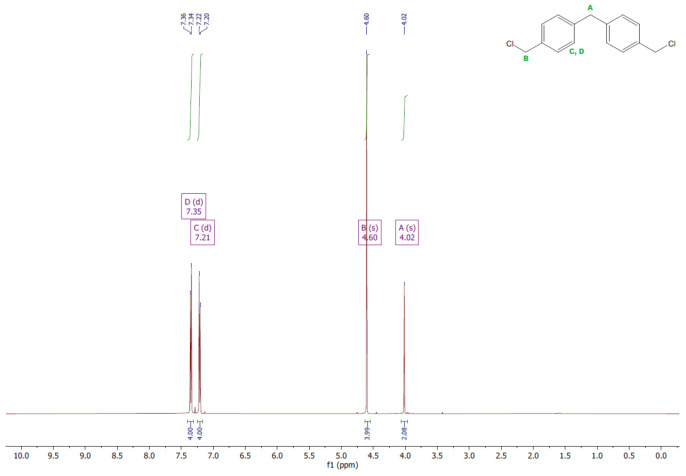
^1^HNMR spectrum of 1,1′-methanediylbis[4-(chloromethyl)benzene].

**Figure 7:**
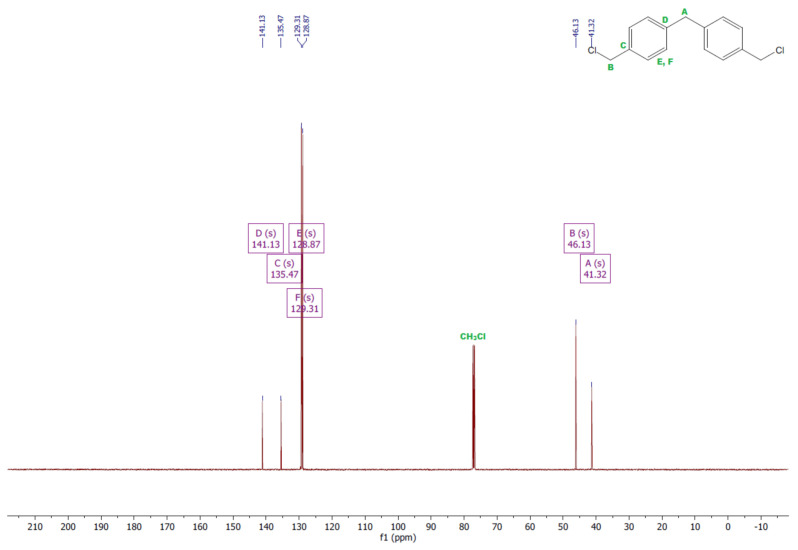
^13^CNMR spectrum of 1,1′-methanediylbis[4-(chloromethyl)benzene].

**Figure 8:**
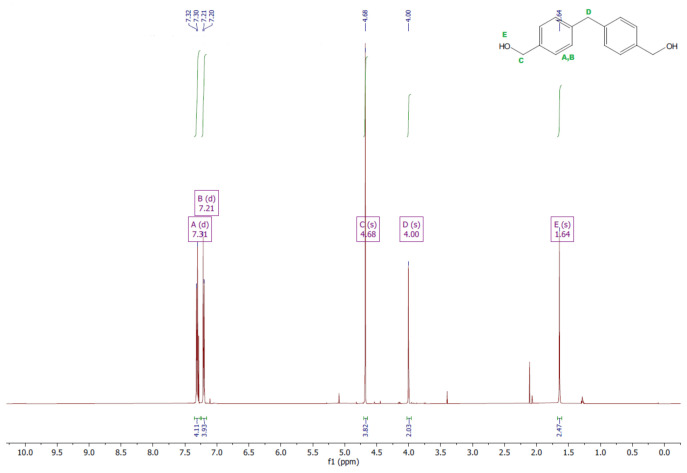
^1^HNMR spectrum of diol M.

**Figure 9:**
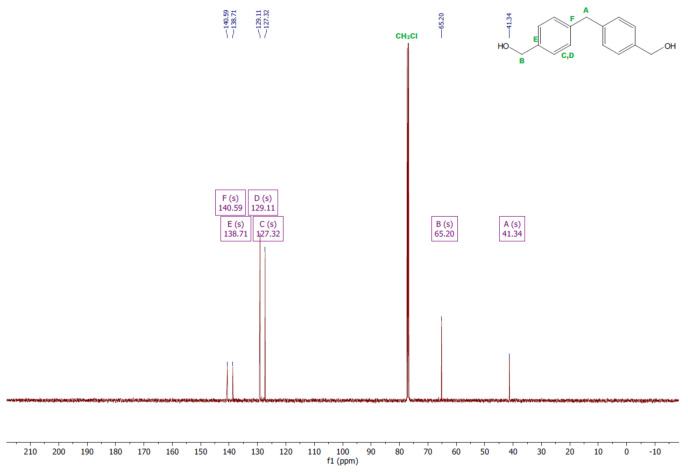
^13^CNMR spectrum of diol M.

**Figure 10:**
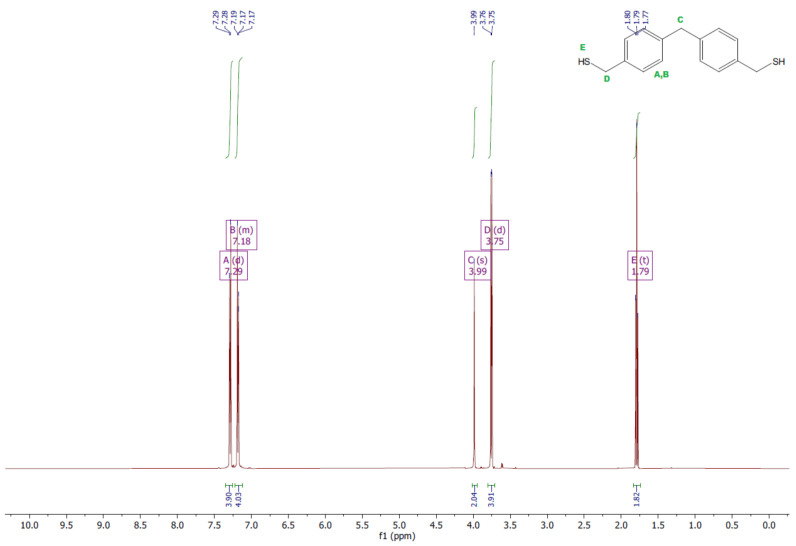
^1^HNMR spectrum of dithiol.

**Figure 11:**
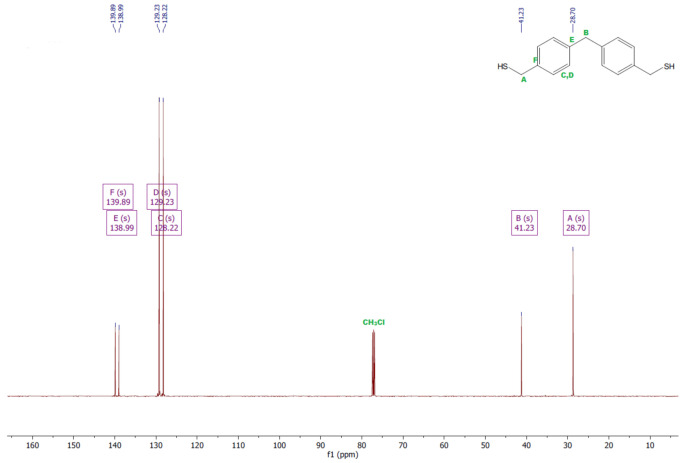
^13^CNMR spectrum of dithiol.

**Figure 12:**
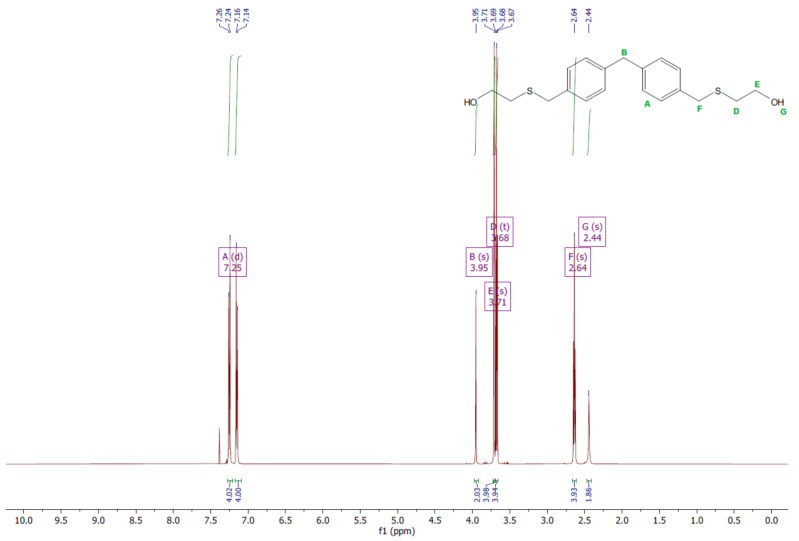
^1^HNMR spectrum of diol E.

**Figure 13:**
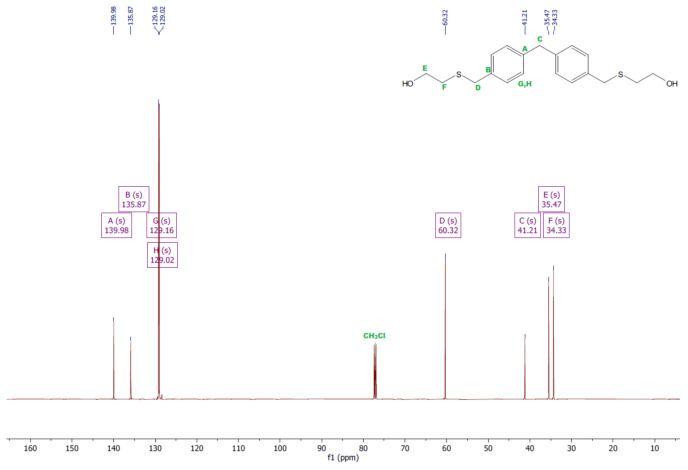
^13^CNMR spectrum of diol E.

**Figure 14:**
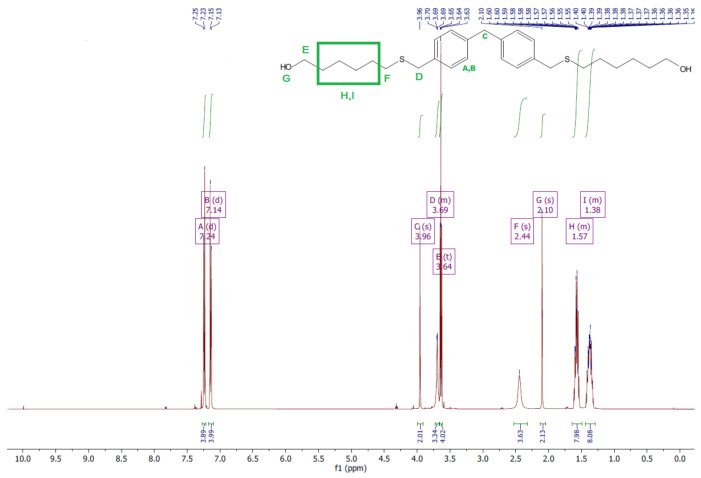
^1^HNMR spectrum of diol H.

**Figure 15:**
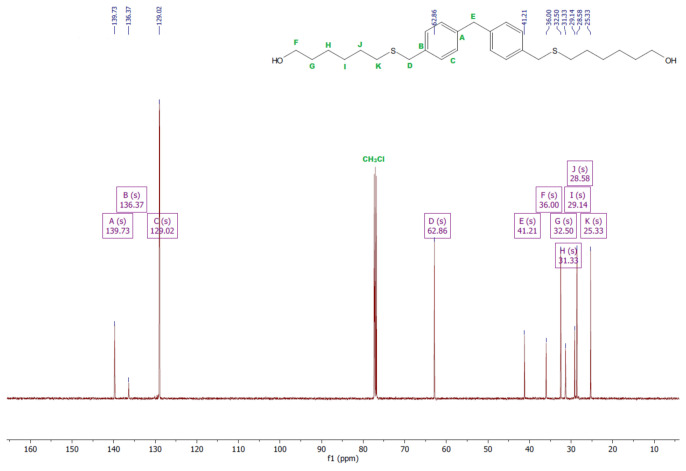
^13^CNMR spectrum of diol H.

The ^1^H and ^13^C NMR analyses were performed for all synthesized monomers and the chlorine derivative of diphenylmethane (1,1′-methanediylbis[4-(chloromethyl)benzene]). NMR spectroscopy was applied in order to determine the monomers’ structures. Deuterated chloroform was used as the solvent. The NMR analysis allowed confirmation of the structures of the obtained monomers. All NMR spectra are presented in Figure 6, Figure 7, Figure 8, Figure 9, Figure 10, Figure 11, Figure 12, Figure 13, Figure 14 and Figure 15. The detailed information about the signals is presented below:

(a) 1,1′-methanediylbis[4-(chloromethyl)benzene]
  ^1^HNMR (500 MHz, CDCl_3_—d, δ ppm): 7.35 (d, J = 8.1 Hz, 4H), 7.21 (d, J = 8.21 Hz, 4H), 4.60 (s, 4H), 4.02 (s, 2H).
  ^13^CNMR (126 MHz, CDCl_3_—d, δ ppm): 141.13 (PhC), 135.47 (CPh), 129.31 (Ph), 128.87 (Ph), 46.13 (Cl-CH_2_-Ph), 41.32 (Ph-CH_2_-Ph).
(b) Diol M
  ^1^HNMR (500 MHz, CDCl_3_—d, δ ppm): 7.31 (d, J = 8.1 Hz, 4H), 7.21 (d, H = 8.21 Hz, 4H), 4.68 (s, 4H), 4.00 (s, 2H), 1.64 (s, 2H).
  ^13^CNMR (126 MHz, CDCl_3_—d, δ ppm): 140.59 (PhC), 138.71 (CPh), 129.11 (Ph), 127.32 (Ph), 65.20 (OH-CH_2_-Ph), 41.34 (Ph-CH_2_-Ph).
(c) Dithiol
  ^1^HNMR (500 MHz, CDCl_3_—d, δ ppm): 7.29 (d, J = 8.1 Hz, 4H), 7.22–7.00 (m, 4H), 3.99 (s, 2H), 3.75 (d, J = 7.5 Hz, 4H), 1.79 (t, J = 7.5 Hz, 2H).
  ^13^CNMR (126 MHz, CDCl_3_—d, δ ppm): 139.89 (PhC), 138.99 (CPh), 129.23 (Ph), 128.22 (Ph), 41.23 (-CH_2_), 28.70 (SH-CH_2_-Ph).
(d) Diol E
  ^1^HNMR (500 MHz, CDCl_3_—d, δ ppm): 7.25 (d, J = 8.1 Hz, 8H), 3.95 (s, 2H), 3.71 (s, 4H), 3.68 (t, J = 6.1 Hz, 4H), 2.64 (s, 4H), 2.44 (s, 2H).
  ^13^CNMR (126 MHz, CDCl_3_—d, δ ppm): 139.98 (PhC), 135.87 (CPh), 129.16 (Ph), 129.02 (Ph), 60.32 (-S-CH2-Ph), 41.21 (Ph-CH_2_-Ph), 35.47 (OH-CH_2_-), 34.33 (-CH_2_-S-).
(e) Diol H
  ^1^HNMR (500 MHz, CDCl_3_—d, δ ppm): 7.24 (d, J = 8.1 Hz, 4H), 7.14 (d, J = 8.1 Hz, 4H), 3.96 (s, 2H), 3.69 (m, 4H), 3.64 (t, J = 6.6 Hz, 4H), 2.44 (s, 4H), 2.10 (s, 2H), 1.68–1.31 (m,16H).
  ^13^CNMR (126 MHz, CDCl_3_—d, δ ppm): 139.73 (PhC), 136.37 (CPh), 129.02 (Ph), 62.86 (-S-CH_2_-Ph), 41.21 (Ph-CH_2_-Ph), 36.00 (OH-CH_2_-), 32.50 (-CH_2_(II)-), 31.33 (-CH_2_(III)-), 29.14 (-CH_2_(IV)-), 28.58 (-CH_2_(V)-), 25.33 (-CH_2_-S-).

### 3.2. ATR–FTIR Analysis

**Figure 16:**
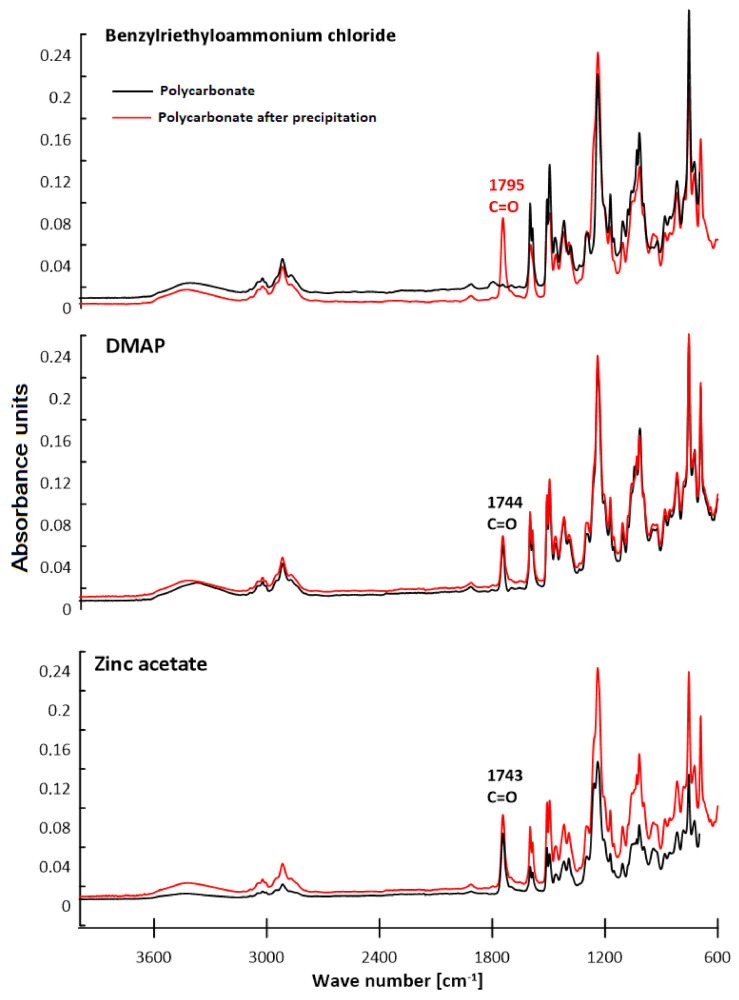
ATR–FTIR spectra of polycarbonates based on diol E.

**Figure 17:**
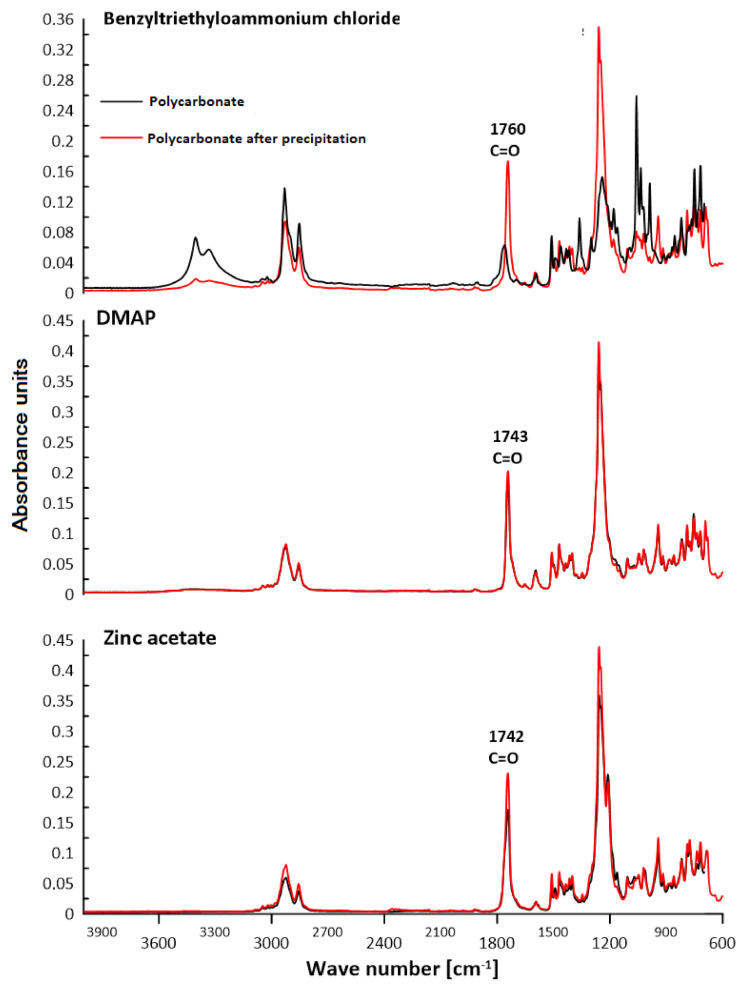
ATR–FTIR spectra of polycarbonates based on diol H.

**Figure 18:**
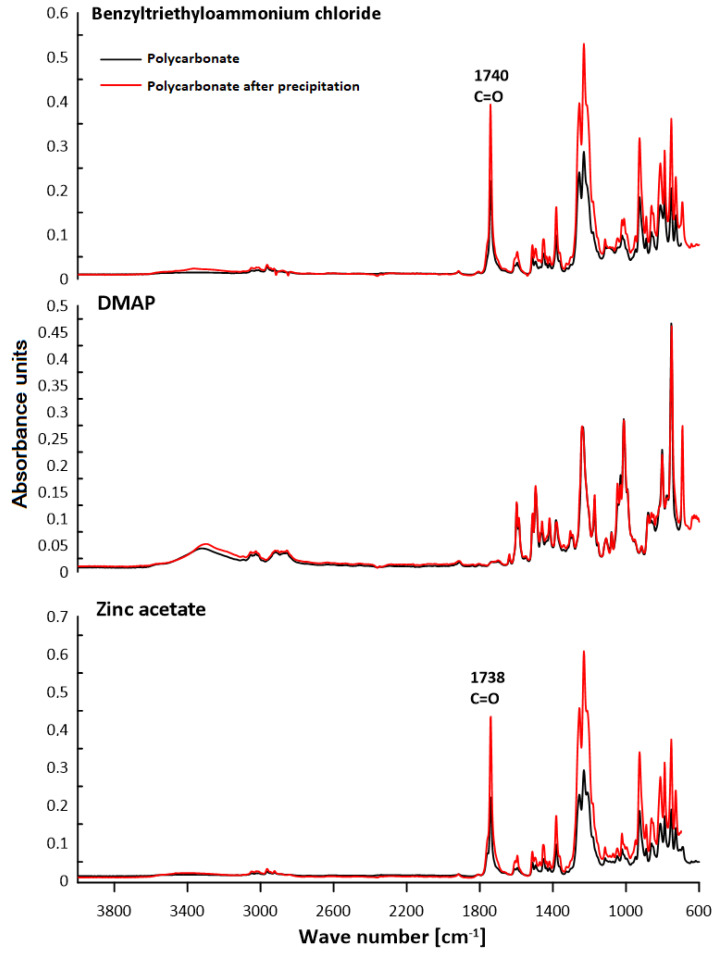
ATR–FTIR spectra of polycarbonates based on diol M.

**Figure 19:**
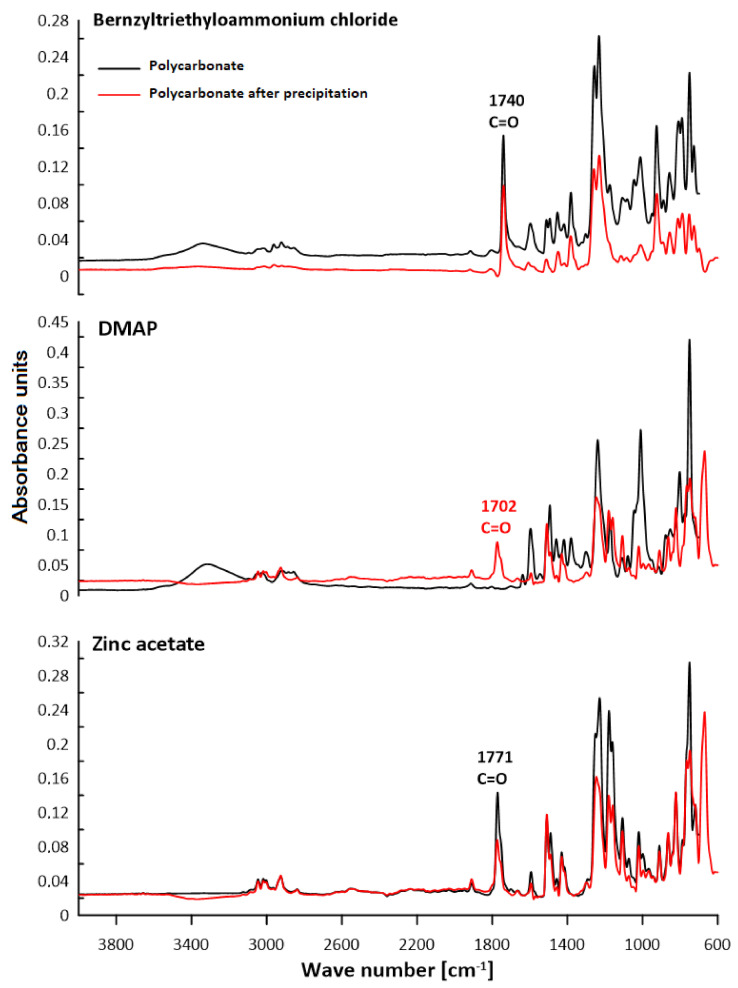
ATR–FTIR spectra of polycarbonates based on dithiol.

The chemical structure of polycarbonates was confirmed by attenuated total reflection–Fourier transform infrared spectroscopy. The study consisted of observing the changes in the positions of the absorption bands for the characteristic functional groups of the obtained polycarbonates. Figure 16, Figure 17, Figure 18 and Figure 19 present the results of the polycarbonates analyses: the pure polycarbonate and the polymers obtained after precipitation from methane dichloride and methanol. The most important vibrations occurring in the spectra are presented in Table 1.

Due to the fact that the main components of the polymers are diphenyl carbonate and the diphenylmethane derivatives, the spectra are similar. The presence of the carbonyl group was considered as evidence of the polycarbonates’ formation. The valence vibrations of the C=O group occur in the range of 1600–1900 cm^−1^. In the aromatic esters this range is narrowed down to 1730–1780 cm^−1^. In our previous studies of composites with polycarbonate as a filler, the carbonyl group peak was even narrower. The signal from the carbonyl group was in the range of 1730–1725 cm^−1^ for all materials [25]. In the present study, this effect was found for all samples except for the polymers: diol E + DPC + benzyltriethylammonium chloride, diol M + DPC + DMAP, dithiol + DPC + DMAP. In these cases, polycarbonates were not formed. The vibrations of the C–O group for saturated esters are visible in the range of 1050–1330 cm^−1^ for all samples. The signal for the hydroxyl group occurs for most samples. This could be related to the presence of the OH group in diols. Another characteristic absorption band in the range of 2962–2915 cm^−1^ is derived from the stretching vibrations from the C–H aliphatic groups. This effect is visible for each composite. Multiple bands ranging from 1596 cm^−1^ to 1449 cm^−1^ can be associated with the vibrations of C–H and C=C bonds related to the benzene rings and aromatic skeletons. These come from both the aromatic diols and dithiol. The peaks around 1400–1450 cm^−1^ originate from the C–H deformation in the -CH_2_- group. The bands around 1295–1012 cm^−1^ could be attributed to C–O stretching vibrations. The signal at 811–988 cm^−1^ was also associated with the C–C vibrations from the aromatic part. The spectra for the polymers obtained after precipitation from methane dichloride and methanol are characterized mostly by greater signal intensity. As for the carbonyl group, it was not visible in the spectra of diol M + DPC + DMAP. To sum up, the presence of the carbonyl group (C=O) appeared in 20 of 24 samples. Our previous studies discussed the effect of sulfur atoms on the infrared spectra [26,27]. The spectra of monomers are included in the Supplementary Materials (Figure S1 and Table S1).

### 3.3. DSC Analysis (Differential Scanning Calorimetry)

**Figure 20:**
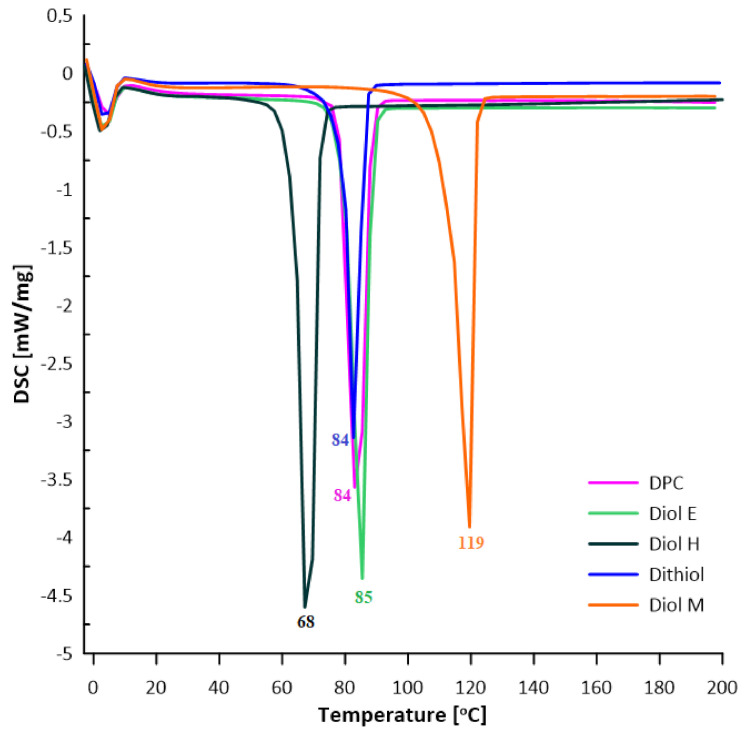
DSC curves of monomers.

The thermal properties of the obtained monomers were studied by means of DSC analysis. Characteristic parameters were determined and collected in Table 2. Monomers were tested in the temperature range 0–200 °C (Figure 20). The DSC analysis allowed the determination of the minimum temperature of the transesterification reaction of the monomers used with DPC.

### 3.4. GPC Analysis

**Figure 21:**
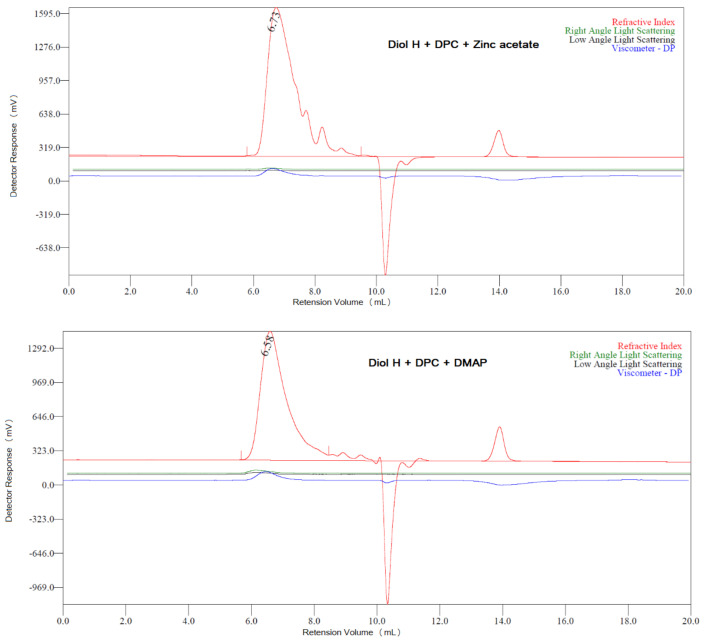
GPC chromatograms for polycarbonates dissolved in THF.

The purpose of the GPC analysis was to determine the molar mass distribution of the obtained polycarbonates. Due to their high chemical resistance (only two polymers completely dissolved in tetrahydrofuran), it was not possible to perform the analysis: the remaining polycarbonates were insoluble or only partially dissolved in THF. The polymers that dissolved in THF (i.e., diol H + DPC + zinc acetate, diol H + DPC + DMAP) were characterized by $\bar{M}$_n_ = 1570 and 6000 g/mol, $\bar{M}$_w_ = 4346 and 11,387 g/mol, and molar mass dispersity (Ð_M_) of 2.768 and 1.898, respectively. Figure 21 shows the chromatograms for these polymers.

The asymmetry of the peaks in the chromatograms indicates the heterogeneity of the molar masses of the polymers (presence of fractions with different molar masses, including oligomers).

## 4. Conclusions

As a result of the multistage synthesis reactions, four aromatic–aliphatic compounds were obtained as diphenylmethane derivatives: diol M, diol E, diol H, and dithiol. The chemical structure of each compound was confirmed by the spectroscopic techniques ^13^C and ^1^H NMR. Additionally, the ATR–FTIR spectra of the compounds are shown. The new polycarbonates were obtained using transesterification reactions and the polycondensation process. Three catalysts were used. The presence of the carbonyl group in the infrared spectrum was used to confirm the formation of the polymer. Taking into account raw materials and comparative materials, the carbonyl group appeared in 20 samples. The polycarbonates were characterized by high resistance to THF. The obtained results of the GPC analysis indicated relatively large molar masses and small dispersity.

In summary, the use of unconventional compounds such as diols resulted in the production of polycarbonates free of toxic BPA.

## Figures and Tables

**Table 1:** Characteristic signals in ATR–FTIR spectra [cm^−1^].

Polymer	C–H Aliph.	C–H Arom.	C=C Arom.	C–O	C=O	–OH
Diol E + DPC + Zinc acetate	2916	881	1596 1463	1239 1172	1743	3426
Diol E + DPC + DMAP	2915	816	1598	1239 1171	1744	-
Diol E + DPC + Benzyltriethylammonium chloride	2916	880	1597	1295 1238 1104	-	3458
Diol H + DPC + Zinc acetate	2923	943	1449	1257 1210	1742	-
Diol H + DPC + DMAP	2929	943	1598 1494	1259	1743	-
Diol H + DPC + Benzyltriethylammonium chloride	2931	988 819	1452	1239 1180 1100	1760	3408
Diol M + DPC + Zinc acetate	2961	924 854	1469	1228	1738	-
Diol M + DPC + DMAP	2917	915 860	1469	1235 1172 1109	-	3319
Diol M + DPC + Benzyltriethylammonium chloride	2929	915 860	1587 1490	1253 1230 1021	1740	-
Dithiol + DPC + Zinc acetate	2958	920 863	1593	1178 1106	1771	-
Dithiol + DPC + DMAP	2960	914 825	1595	1238 1172 1119	-	3315
Dithiol + DPC + Benzyltriethylammonium chloride	2962	925 811	1596	1256 1231 1174	1740	3370

**Table 2:** DSC data of monomers.

Monomer	T_m_ [°C]	ΔH_m_ [J/g]
Diol E	85	143
Diol H	68	161
Diol M	119	154
Dithiol	84	101
DPC	84	134

## Data Availability

The data presented in this study are available on request from the corresponding author.

## Supplementary Materials

The following are available online at https://www.mdpi.com/article/10.3390/polym13244437/s1, Figure S1: ATR–FTIR analyses of monomers; Table S1: Characteristic signals in ATR–FTIR spectra of monomers [cm^−1^].
