# Supplementary material for: Synthesis and Spectroscopic Analyses of New Polycarbonates Based on Bisphenol A-Free Components
Source: Polymers (Basel). 2021 Dec 17;13(24):4437. doi: 10.3390/polym13244437 (PMC8704700; doi:10.3390/polym13244437)
Supplement: Supplementary file 1 [file polymers-13-04437-s001.zip › polymers-1494546-supplementary.pdf]

polymers

MDPI

Supplementary Materials

Synthesis and Spectroscopic Analyses of New Polycarbonates Based on Bisphenol a-Free Components

Krystyna Wnuczek [1,*], Andrzej Puszka [1] and Beata Podkościelna [1]

[1] Department of Polymer Chemistry, Faculty of Chemistry, Institute of Chemical Sciences, Maria Curie-Skłodowska University, Gliniana 33, 20-614, Lublin, Poland
* krystyna.wnuczek@poczta.umcs.lublin.pl

Citation: Wnuczek, K.; Puszka, A.; Podkościelna, B. Synthesis and Spectroscopic Analyses of New Polycarbonates Based on Bisphenol A-Free Components. *Polymers* **2021**, *13*, 4437. https://doi.org/10.3390/polym13244437

Academic Editors: Andrzej Puszka, Beata Podkościelna and Dan Rosu

Received: 19 November 2021
Accepted: 13 December 2021
Published: 17 December 2021

Publisher's Note: MDPI stays neutral with regard to jurisdictional claims in published maps and institutional affiliations.

Copyright: © 2021 by the authors. Licensee MDPI, Basel, Switzerland. This article is an open access article distributed under the terms and conditions of the Creative Commons Attribution (CC BY) license (http://creativecommons.org/licenses/by/4.0/).

S2.3 Synthesis of monomers

S2.3.1 Synthesis of 1,1′-methanediylbis[1-(chloromethyl)benzene]

225g (1.33 mol) of diphenylmethane, 92g (1.02 mol) of paraformaldehyde, 320g (2.24 mol) of anhydrous 98% zinc chloride and 320 cm³ of pure acetic acid, 140 cm³ of concentrated 36% hydrochloric acid and 400 cm³ of cyclohexane were added, and then while stirring, gaseous hydrogen chloride was added at such a rate that the temperature of the mixture increased by 0.5°C for a minute. Under these conditions, the contents of the flask turned transparent after about 2 hours and reached a temperature of about 50°C. The further course of the reaction did not provide enough heat to maintain this temperature, so hydrogen chloride was passed through while heating the contents of the flask in a water bath at 57-62°C. After 3 hours the reaction was completed and after stopping the flow of hydrogen chloride, the flask contents split into two liquid phases, the upper, lighter, main product having the appearance of a greenish oil. After cooling the contents of the flask to room temperature, the layer was easy to filter. After the mass was filtered off, washed with water and compressed, there was obtained a crude product which was washed twice with 5% Na_2CO_3 solution, 500 cm³ each, and twice with methanol, 160 cm³ each. 160g of product was obtained.

S2.3.2 Synthesis of (methanediyldibenzene-4,1-diyl)dimethanethiol (Ditiol)

In a 1000 cm³ round bottom flask, 122g (0.46 mol) of 1,1′-methanediylbis[4-(chloromethyl) benzene] was dissolved in 450 cm³ of 1,4-dioxane and after the addition of 71g (0.93 mol) of thiourea at a reflux for 1 hour. The isolated precipitate of the isothiourea salt was filtered off cold. 800 cm³ of a 12% aqueous NaOH solution was added and refluxed for 1 hour under a water-jacketed reflux condenser. The solution was filtered hot and the cooled filtrate acidified with dilute hydrochloric acid (1:1). A fine crystalline precipitate separated from the solution left at room temperature was filtered off, and then purified by dissolving 10% aqueous NaOH twice in 800 cm³ and precipitating with the 5% HCl solution. The crude product was purified by crystallization from ethanol (1g per 10 cm³ of the solvent).

S2.3.3 Synthesis of 2,2′-[methanediylbis(benzene-4,1-diylmethanediylsulfanediyl])diethanol (Diol E)

30g (0.12 mol) of dithiol was dissolved in 500 cm³ of a 10% aqueous NaOH solution, then the solution was transferred to a 1000 cm³ three-necked flask equipped with a mechanical stirrer, water jacket reflux condenser and dropping funnel. 20.33g (0.25 mol) of 2-chloroethanol was added via a dropping funnel. As the reaction proceeded, a white precipitate of diol E appeared. The dropping funnel was washed with 10 cm³ of ethanol. The whole was heated in a boiling water bath for 1.5 h. After cooling, the separated precipitate was filtered off on a funnel and washed with distilled water until becoming neutral. The crude product was purified by recrystallization twice from benzene (120 cm³). 26g of diol E was obtained.

S2.3.4 Synthesis of 6,6'-(methanediyl(benzene-4,1-diylmethanediylsulfanediyl)dihexan-1-ol (Diol H)

30g (0.12 mol) of dithiol was dissolved in 500 cm^3 of a 10% aqueous NaOH solution, then the solution was transferred to a 1000 cm^3 three-necked flask equipped with a mechanical stirrer, water jacket reflux condenser and dropping funnel. Using a dropping funnel, 35.95g (0.25 mol) of 6-chlorohexan-1-ol was added in small portions. As the reaction progressed, a white precipitate of diol H appeared. After the dropwise addition, the funnel was washed with 10 cm^3 of ethanol, and then the contents of the flask were heated in a boiling water bath for 1.5h. After cooling, the contents of the flask with the separated product were filtered on a funnel, washed with distilled water until becoming neutral and dried. The crude product was crystallized from benzene (150 cm^3) to obtain 50g of diol H.

S.3 Results and discussion

S3.2 ATR-FTIR analysis

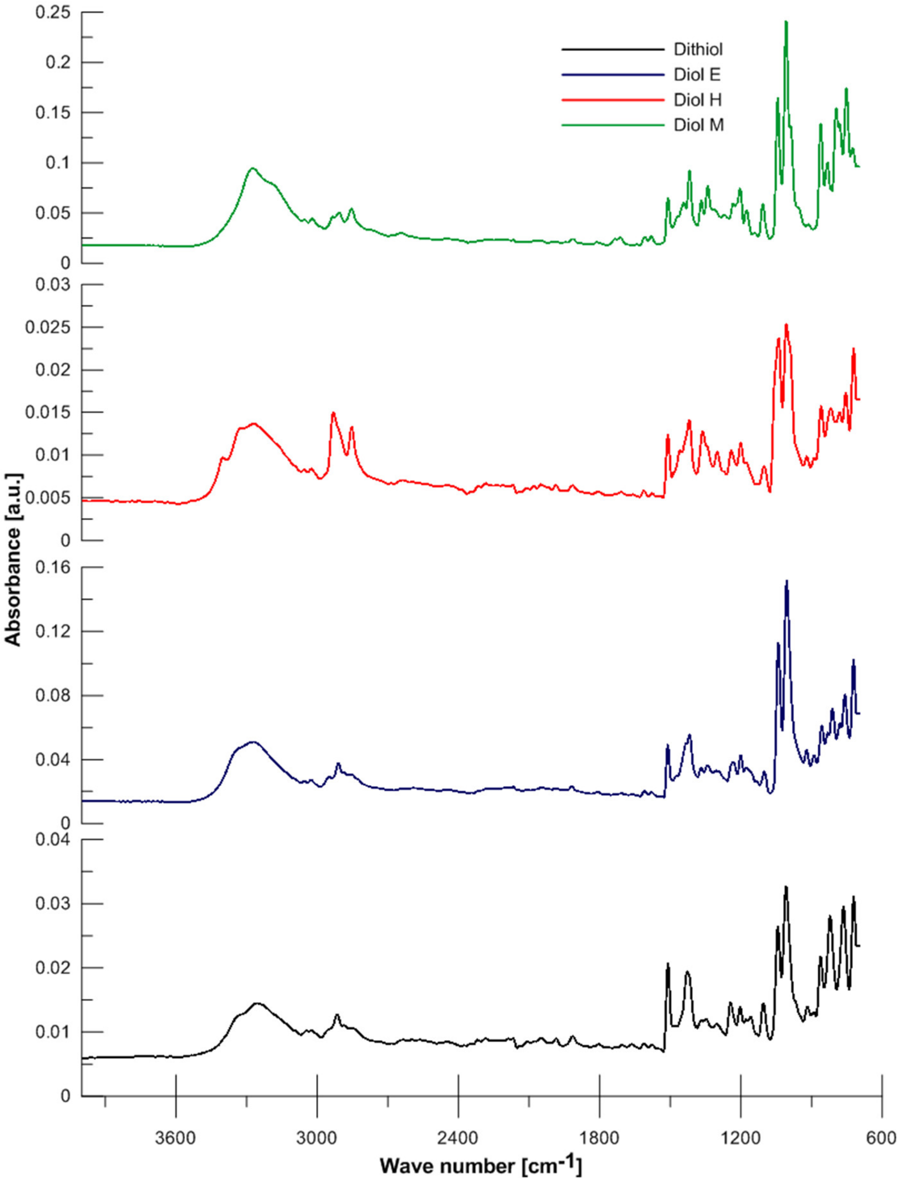

Figure S1. ATR-FTIR analyses of monomers.

Table S1. Characteristic signals on ATR-FTIR spectra of monomers [cm^{-1}].

Monomer	-OH	Ar-H	$-CH_2-$	-C-H-	-C-O-	C-C-	R-Ar-R	$-S-CH_2-$
Dithiol	-	3256<br>3023<br>1914<br>721	2915<br>1368	1203	1105<br>1043	1243	1509<br>861<br>762	1427<br>1008<br>917
Diol E	3270	889<br>1510<br>2910	721<br>1417	110<br>1200<br>1312	1039<br>1234<br>1340	811	758	920<br>1005
Diol H	3269	858<br>1510<br>2931	721<br>1419	1100<br>1200<br>1300	1041<br>1240<br>1362	818	780	1006<br>2853
Diol M	3274	3021<br>860	2906<br>725	2853<br>1203<br>1007	1043<br>1107<br>1340<br>1368<br>1417	1175	795<br>751	-
